# 

**Published:** 1867-03

**Authors:** 


### CONTENTS FOR MARCH.

ORIGINAL CONTRIBUTIONS.

Extirpation of the Entire Thyroid Gland. By E. L. Marshall, M.D., 97
Fifteen Cases of "Cerebro-Spinal Meningitis." By Chas. M. Clark, M.D., 104
Death from Hemorrhage. By G. B. Lester, M.D., 112
The Medical Aspect of Iowa. By A. G. Field, M.D., 114

PROCEEDINGS OF MEDICAL SOCIETIES.

Morgan County Medical Society, 122
The Iowa and Illinois Central District Medical Association, 124
Dr. Brown's Lecture before the Macon County Medical Society, 125

EDITORIAL, 134

Valedictory, 134
The Editor's Salutation, 134
*Book Notices*—The Science and Practice of Medicine. By William Aitken, M.D., Edin., Professor of Pathology in the Army Medical School, and formerly Demonstrator of Anatomy in the University of Glasgow, etc. In two volumes. Vol. I. 8vo. pp. 955. From the Fourth London Edition, with Additions by Meredith Clymer, M.D., Late Professor of the Practice of Medicine in the University of New York, 136
To Correspondents, 138
Appointment, 139
Rush Medical College, 140
Chicago College of Pharmacy, 142
American Medical Association, 143

